# Recognition of Gait Patterns in Older Adults Using Wearable Smartwatch Devices: Observational Study

**DOI:** 10.2196/39190

**Published:** 2022-08-24

**Authors:** Hyeon-Joo Kim, Hyejoo Kim, Jinyoon Park, Bumjo Oh, Seung-Chan Kim

**Affiliations:** 1 Machine Learning Systems Lab College of Sports Science Sungkyunkwan University Suwon Republic of Korea; 2 Department of Family Medicine Seoul Metropolitan Government - Seoul National University Boramae Medical Center Seoul Republic of Korea

**Keywords:** activity recognition, machine learning, health monitoring, gait analysis, wearable, sequence classification, mobile health, mHealth, neural network

## Introduction

It is challenging to routinely assess gait unless dedicated measuring devices are available. Inspired by a recent study that reported high classification performance of activity recognition tasks using smartwatches [1], we hypothesized that the recognition of gait-related activities in older adults can be formulated as a supervised learning problem. To quantify the complex gait motion, we focused on hand motion because disturbed hand motions are frequently reported as typical symptoms of neurodegenerative diseases [2].

## Methods

### Data Acquisition

**Figure 1:**
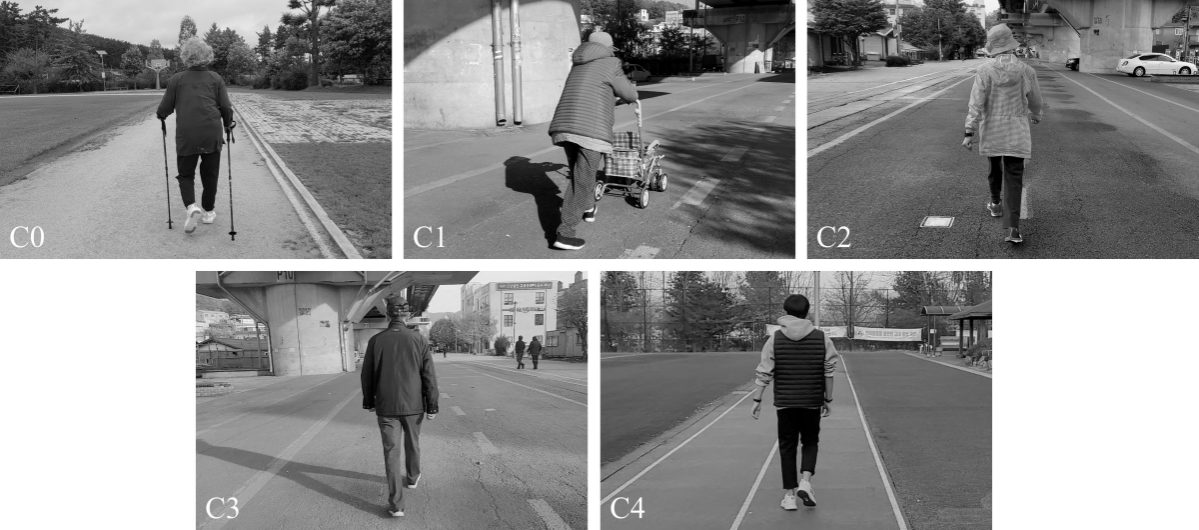
Five different gait styles: cane-assisted gait (C0), walker-assisted gait (C1), gait with disturbances (C2), gait without disturbances (C3), and gait without disturbances in young controls (C4).

We recruited 39 older adult participants (age: 80.4, SD 6.5 years; n=38, 73.7% women) from a local community. The number of participants for each class was as follows: cane-assisted gait (C0) (n=7), walker-assisted gait (C1) (n=5), gait with disturbances (C2) (n=21), gait without disturbances (C3) (n=6), and gait without disturbances in young controls (C4) (n=12). During the experiment, participants were asked to wear a smartwatch (DW9F1; Fossil Group, Inc) on each wrist and walk at a normal speed similar to their usual walk. Figure 1 shows example photographs taken during the experiment.

### Classification

The multivariate time-series (MTS) signals captured at a sampling rate of 50 Hz were segmented into 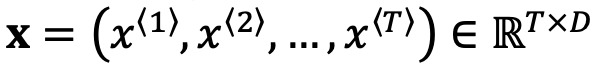. Here, 
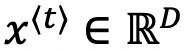
 represents the inertial motion at a specific moment, *t*.  In this study, *D* was 12 (=6×2), since each smartwatch measures the 6-DOF (6 degrees of freedom) motion separately, and *T* was 100 (approximately 2s) so that each **x** could contain at least a full gait cycle. The task in our study was to infer the type of gait activity, 
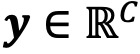
, where *C* was 5. Our neural network systems, tailored to learn gait features from MTS data, were trained in an end-to-end fashion using state-of-the-art deep learning architectures, including Conv1D [3], long short-term memory (LSTM) [4], and an LSTM with an attention mechanism [5].

### Ethics Approval

All participants were enrolled after institutional review board (IRB) approval (Sungkyunkwan University IRB approval number: SKKU 2021-12-014).

## Results

**Figure 2:**
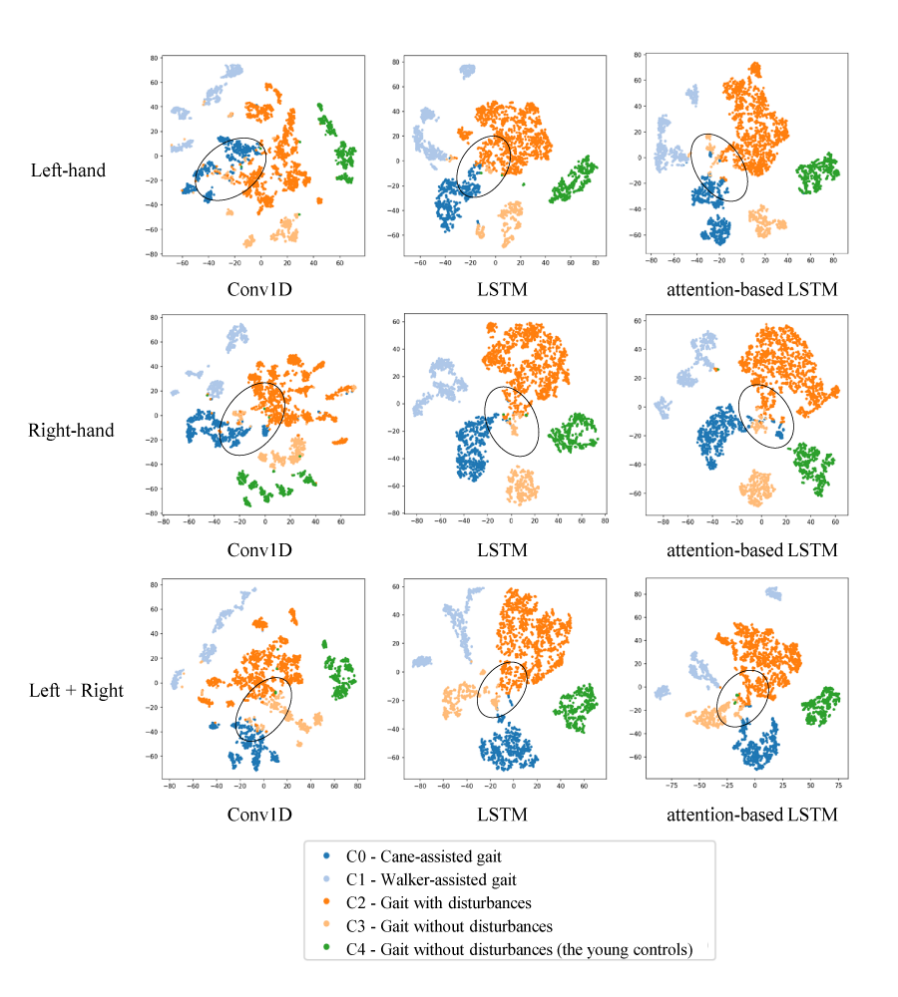
Feature visualization using t-distributed stochastic neighbor embedding. Each point is colored according to the predicted class. LSTM: long short-term memory.

We employed the accuracy and macro average of the *F*_1_-score, *F_m_*, as a measure of performance. For the both-hands condition, the accuracy (*F_m_*) was 0.9757 (0.9728), 0.9736 (0.9699), and 0.9771 (0.9738) when Conv1D, LSTM, and attention-based LSTM were employed, respectively. In the case of the left-hand and right-hand conditions, the accuracies (*F_m_*) obtained in the left-hand condition were 0.9652 (0.9623), 0.9611 (0.9583), and 0.9630 (0.9592), respectively. In the right-hand condition, the accuracies (*F_m_*) were 0.9724 (0.9706), 0.9673 (0.9643), and 0.9673 (0.9635) for the same employed models, respectively. We also examined the learned representations as shown in Figure 2 using t-distributed stochastic neighbor embedding (t-SNE) [6], which visualizes the high-dimensional vectors by projecting them into a 2D space in such a way that similar points cluster together.

## Discussion

The experimental results demonstrated an acceptable classification performance (ie, both accuracy and the *F_m_* score were higher than 0.95). However, there is systematic confusion, such as recognizing C3 as C2 (0.03-0.04 for the left hand, 0.05-0.07 for the right hand, and 0.05-0.06 for both hands, respectively) as shown in Figure 2 (see the region highlighted in black). It is noteworthy that the classification performance of the single-hand condition was similar to that of the both-hands condition, suggesting that wearing a single smartwatch is sufficient for the proposed gait assessment task. From the t-SNE plot, it was found that points from the LSTM and attention-based LSTM exhibit a more clustered distribution than those from the Conv1D model. We expect that the proposed approach can be applied to various health care applications for older adults (eg, wearable detection of gait disturbances).

## Abbreviations

6-DOF: 6 degrees of freedom
LSTM: long short-term memory
MTS: multivariate time-series
t-SNE: t-distributed stochastic neighbor embedding
